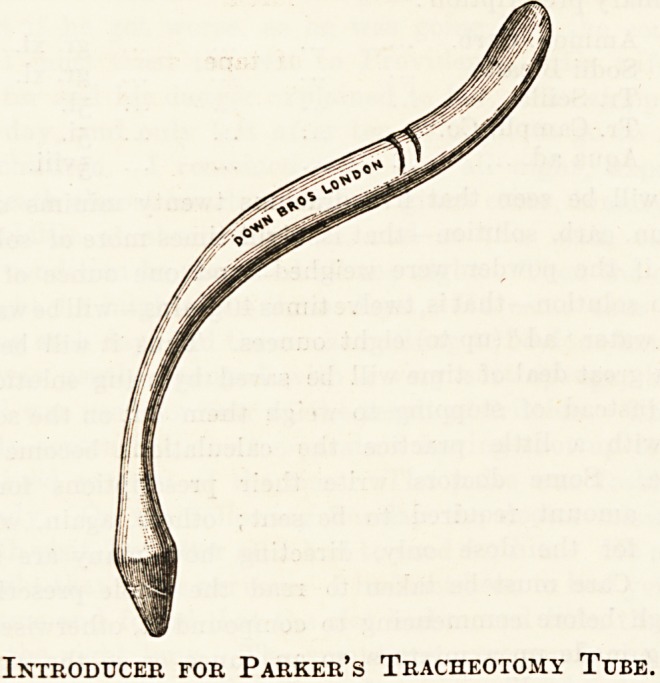# The Hospital. Nursing Section

**Published:** 1905-11-18

**Authors:** 


### The Hospital.

**Nursing Section.**

Contributions to "THE HOSPITAL," should be addressed to the EDITOR, 'THE HOSPITAL" NURSING SECTION, 28 & 29 Southampton Street, Strand, London, W.C.

No. 999.—VOL. XXXIX. SATURDAY, NOVEMBER 18, 1905.

### Notes on News from the Nursing World.

### THE QUEEN'S FUND FOR THE UNEMPLOYED.

WE are not surprised to have received from several of our readers expressions of a desire to contribute to the Queen's Fund for the Unemployed, and suggesting that we should institute a direct appeal to nurses throughout the country to support it. It is natural enough that Her Majesty's splendid action should awaken the sympathy of a class who are brought so directly into touch with the poor as the nurses in our great hospitals. But we know that the means of nurses are usually as slender as their generosity is spontaneous; and we think that if they have any money to spare, their first duty is to the unemployed of their own class, their sisters in distress, for whose benefit the Benevolent Fund of the Royal National Pension Fund for Nurses was founded.

### CONFERENCE OF REPRESENTATIVES OF DISTRICT ASSOCIATIONS.

A CONFERENCE was held last week of representatives of the different nursing associations affiliated to Queen Victoria's Jubilee Institute for Nurses. One subject of discussion was school nursing in the elementary schools, the council of the Institute having recently circulated a memorandum advocating the employment of Queen's nurses under the education authorities to attend to the children suffering from minor ailments. The question of better co-operation between hospitals and district nursing associations in the matter of out-patients was also dealt with, and a resolution was passed that further inquiry should be made into the methods existing at present, with a view to bringing the two branches of nursing into more practical and systematic relations. It was decided that in future the conference should meet twice a year.

### THE NEW MATRON OF THE LONDON HOMŒOPATHIC HOSPITAL.

AT a meeting last week of the Board of Management of the London Homœopathic Hospital, Great Ormond Street, Bloomsbury, Miss Victoria Daunt was elected lady superintendent in succession to Miss M. Brew, whose resignation we lately announced. It is rather remarkable that the choice of the Board should have fallen upon Miss Daunt, who, since the appointment of Miss Vernet to the Middlesex Hospital, has been acting matron at the National Hospital for the Paralysed and Epileptic, also in Bloomsbury. She has thus only to step across the way in order to take up her new duties. There were a great many applicants for the vacancy, and it speaks well for the Great Northern Central Hospital, where Miss Daunt was trained, that she was selected from among them. She has been successively theatre sister, sister of the women's surgical and gynæcological ward, and sister of the men's ward at that institution, from which she went to the National Hospital as night sister, subsequently becoming housekeeper sister. We hope that under the auspices of the new lady superintendent the nursing staff of the London Homœopathic Hospital will soon obtain better sleeping accommodation. Miss Brew, who retires on a well-earned pension, worked hard in the interests of the charity, but her successor will find ample scope for her abilities.

### ONE BEDROOM FOR THREE NURSES.

EVERY nurse ought to have a bedroom to herself. At Portsmouth Parish Infirmary three nurses have to sleep in a room where there is only accommodation for two. This is a state of affairs which should be altered at once, and we trust that the Portsmouth Guardians will lose no further time in acting upon the recommendation of Mr. Baldwin Fleming, the Local Government Board Inspector who has urged them to extend the nurses' home. This recommendation was made in 1904, and even consideration for the pockets of the ratepayers does not absolve the Guardians from providing adequate sleeping accommodation for the nurses in their employ.

### MATRON'S POUND DAY AT DOVER HOSPITAL.

FOR the second time the matron of the Royal Victoria Hospital at Dover has held her pound day with great success. The sum received in money was £68 19s. 6d., and upwards of 3,000 lbs. of goods were brought to the hospital during the day. The latter included 631 lbs. of different varieties of sugar, 383 lbs. of potatoes, 259 lbs. of different kinds of rice, 259 lbs. of soap, 214 lbs. of tea, 112 lbs. of wood, 101 lbs. of jam, 100 lbs. of cornflour, 83 lbs. of flour, and 63 lbs. of cocoa; while among the fruit, represented in numerous lbs., were apples, bananas, dates, figs, grapes, pineapples, and prunes. Vegetables, butter, and other useful commodities were also contributed by many. Even the animals were not forgotten, one of the donations being a bag of dog-biscuits for "Hospital Jack," and a large cod's head for the cats.

### AN IMPRACTICABLE PROPOSAL.

A SUGGESTION is made by a correspondent that in order to enable girls going abroad or young married women to acquire some knowledge of the care of babies in sickness and in health the matrons of some of the children's hospitals should either agree to give a course of simple lectures to those willing to pay for them or allow lady pupils to come into the wards from 10 to 6 with the object of learning the nursing of infants for a monetary consideration. This is quite impossible. Already the matrons of most of our Children's Hospitals have their time so fully occupied that they could not find leisure to lecture to outsiders, the lectures being, of course, valueless to the acting probationers who are obliged to have a more comprehensive curriculum. There are also insurmountable obstacles to admitting daily pupils to work in the wards. But there are not such serious difficulties in the way of girls anxious to gain knowledge of children, as our correspondent imagines. Many of the London hospitals still receive paying probationers for short periods, as well as some of the provincial and cottage hospitals, and in addition there are several institutions throughout England where girls can be received for a few months and be trained exclusively in nursing children, both well and ill. Here they have to do practical work, one month of which would be of more assistance to them than hundreds of words of wisdom addressed to unprepared minds.

### PIONEER CHILDREN AT ASHTON-UNDER-LYNE.

The most interesting feature in connection with the bazaar just held at Ashton-under-Lyne, in aid of the District Nursing Association, was its opening on the last day in the name of the children by the Mayoress. Previous to the ceremony, however, a long procession of girls and boys presented their offerings, the amount collected between them being nearly £75. The Mayoress stated that this amount had been obtained by the little people by the expenditure of much energy and possibly a little self-sacrifice, and she trusted that when they grew up to manhood and womanhood they would still show their sympathy for the suffering poor, and retain the interest they had manifested that day in the Association. We congratulate the children of Ashton-under-Lyne upon the marked success of their efforts, and we hope that they will prove to be the pioneers of many similar undertakings in the country. It is alike good for charity and good for children that their active aid should be enlisted on its behalf.

### ANOTHER VICTIM OF TYPHOID FEVER.

The frequency with which nurses fall victims to typhoid fever is deplorable. The latest instance is that of Miss Gibbon, one of the nurses who volunteered her services in connection with the epidemic of typhoid at Penarth. She had assisted in nursing eighteen cases at the Penarth Isolation Hospital, and was then herself taken ill. As she was only twenty-five years of age she could not have been fully trained. It does not, of course, follow that she did not observe the proper precautions against infection. But we cannot insist too strongly upon the importance of typhoid patients being nursed by those who fully understand that they are liable to take the disease unless the utmost care is exercised in every detail. The fact that probationers in large hospitals who only assist under supervision of a trained sister in nursing typhoid patients, rarely contract the malady, is significant.

### UNTRAINED NURSES AT BRIGHTON WORKHOUSE INFIRMARY.

It will be observed that in the appointments of two staff nurses to the Workhouse Infirmary at Brighton, which we announce to-day, no mention is made of their training. The fact is that, although they have both had nursing experience, neither of them was trained. It was only the other day that the Brighton Guardians refused to replace their probationers by staff nurses because of the increase in cost which the change would involve. There was something to be said in favour of this decision, but there is nothing to be said in support of the employment of untrained staff nurses in an institution where probationers are supposed to be trained.

### THE ENCOURAGEMENT OF OPEN WINDOWS.

The new member for Hampstead was the principal speaker at the third annual meeting of the Kilburn and West Hampstead Nursing Association. Mr. Fletcher, M.P., not only commended the work of the nurses because of their direct ministrations to the sick, but also in the matter of the advice they give to the poor, "and especially in the encouragement of open windows." We are glad that this point was mentioned, for it is one of great, though often unrecognised, importance. Too many of the windows of small houses are hermetically sealed, at any rate from October to April. By constantly urging the necessity of fresh air, the district nurse can do much to induce people to abandon this germ-breeding and vicious habit. The window of every room should be open, at all events for a portion of the day, and the bedroom window should be always kept open during the night. We have no doubt that district nurses are frequently tempted to give up advising in sheer despair; but nothing short of persistence can overcome the prejudice in favour of the closed window.

### QUEEN ALEXANDRA'S MILITARY NURSING SERVICE.

We are officially informed that Miss M. D. Woodhouse, staff nurse in Queen Alexandra's Imperial Military Nursing Service, has been posted to Royal Herbert Hospital, Woolwich. Miss A. M. Pagan, sister, has been posted to Pretoria, and Miss A. A. Wilson, sister, to Middelburg, Cape Colony, on their arrival from England. Miss F. M. MacGregor, staff nurse, has been posted to Middelburg, Transvaal, on her arrival from England. Miss A. M. MacCormac, staff nurse, has been asked to hold herself in readiness for service abroad.

### PRINCESS HENRY OF BATTENBERG AND THE GIPSY CHILD.

The sum of money realised by the bazaar last month in aid of Darlington Hospital is £8,260. The stall of the Mayoress came out first with £407 11s. 7d., and the Old Patients' stall next with £254 1s. 4d. As may be imagined, this splendid result was not achieved without a great deal of hard work, of which the lion's share was borne by the matron, Miss Hunt, and the nursing staff. The matron told Princess Henry of Battenberg, by whom the bazaar was opened, that a gipsy child who had a broken thigh, a dislocated shoulder, and was brought in suffering from concussion of the brain, desired to curtsey to her. The Princess smilingly assented, and the little maiden, attired in a pink cotton dress, walked up very prettily and with a profound curtsey presented a bunch of pink roses to Her Royal Highness, who looked very pleased and said: "Oh, that is very nice."

### MEMOIR OF MISS LOCH, R.R.C.

We understand that a memoir of Miss Catharine Grace Loch, R.R.C., Senior Lady Superintendent of Queen Alexandra's Military Nursing Service for India, written by Surgeon-General A. F. Bradshaw, who was Principal Medical Officer to the forces in India, will shortly be published by Mr. Henry Frowde. The introduction is contributed by Field-Marshal Earl Roberts, who, as a soldier, pays a graceful tribute to the memory of Miss Loch.

### ROYAL NATIONAL PENSION FUND.

At a meeting of the Kingston Infirmary Nurses' League on November 1 Mr. Louis Dick, Secretary of the Royal National Pension Fund for Nurses, gave an interesting address on the work of the Fund. He was listened to with great attention by the large assembly of League members and friends. Invitations to the meeting had been also issued to the matrons and nurses of various institutions in the surrounding district, but, owing to the unfortunate weather, many were unable to be present.

### LADY DUFFERIN ON DISTRICT NURSING.

Both the Dowager Marchioness of Dufferin and Lady Hermione Blackwood were present at the annual meeting of the Armagh District Nursing Association. The former, in moving the adoption of the reports, having referred to the excellent work done by the nurses, the beautiful home in which they live, and the satisfactory state of finance, assured her audience that these things, "would make most district nursing committees green with envy and jealousy." Speaking of nursing in the abstract, she said that if they looked back to the days of Dickens and the Crimean War they would see what progress ladies had made in nursing, and they would realise how capable women were of seeing their own defects and remedying them. The more she saw of district nursing the more splendid work she thought it was, and she felt that there was no better way of alleviating suffering and bringing some comfort into the homes of the sick poor. They all knew the great help a skilled nurse was in their own homes at the time of sickness, and if the nurse could not do away with all suffering she made them feel that "if we cannot lie aisy, she will help us to lie as aisy as we can."

### IRISH NURSES' ASSOCIATION.

An interesting lecture on "The History of Vaccination" was given to the members of the Irish Nurses' Association last week at their rooms in Dublin by Dr. Kirkpatrick, of Steevens' Hospital. Mrs. Manning, Lady Superintendent of the Dental Hospital, presided. Dr. Kirkpatrick illustrated his useful lecture, by many fine lantern-slides, the preventive results of the present system of vaccination. Miss Haughton, Lady Superintendent of Sir P. Dun's Hospital, proposed, and Miss Roberts seconded, a vote of thanks to him, which was adopted with acclamation.

### THE PERILS OF IMPERFECT PROTECTION.

The importance of attention to apparent trifles was illustrated in the evidence given at an inquest held at Fulham on Saturday on a patient who was burnt to death at a private mental asylum at West Kensington. The fire occurred in the deceased's room during the temporary absence of the nurse. A housemaid, having finished her work in the apartment, went elsewhere. Upon returning, ten minutes later, she found the deceased crouching in a corner with her clothes burning and the bedding on fire. The Superintendent of the institution said that in this particular case there was no need for special supervision, but that the instructions to the nurses were definite that they should never leave their patients alone. There was a fire in the room in question protected by a revolving guard. The latter, however, was imperfect and could be opened by a push. The nurse in charge, who had been in the house for sixteen years, and of whom the matron spoke well, stated that she found the guard half open when she was called and believed that the patient set her dress on fire whilst warming her feet on the bars. The jury added a rider to their verdict of "Accidental death," that they considered that the guards should be properly secured.

### FOOTBALL AND NURSING.

An interesting fact in connection with the Loughborough Nursing Association, which has just had its ninth annual meeting, is that the Loughborough Corinthian Football Club contributes substantially to the funds, and that 90 per cent. of the members of the club are working-men. This is but one of several indications that the labours of the Queen's Nurses are fully appreciated by the working-classes in Loughborough. The financial statement, which shows a balance in hand, is of a satisfactory character, and the Mayor, who presided at the meeting, said that he believed more good was done at a small cost by the Nursing Association than by any other society in the town. The nurses attended 154 patients and paid 4,540 visits. The inspector of the district describes their work as "very satisfactory."

### TRAGEDY IN A NURSING HOME.

A shocking tragedy occurred at Brighton a few days ago in a private nursing home. It transpired in the course of evidence before the magistrates, who had the case under investigation on Saturday, that a nurse, described as being from London and having broken down under her training, had suddenly cut the throat of a baby four months old, a patient in the home. The accused was committed for trial, and medical witnesses testified that the crime was committed during sudden mental aberration.

### OUR CHRISTMAS DISTRIBUTION.

We have received a contribution to our Christmas distribution from Nurse Morley and Mrs. Morley, Brockley. All parcels should be addressed to the Editor, 28 and 29 Southampton Street, Strand, London, W.C., with "Clothing Distribution" written on the outside.

### The Nursing Outlook.

"From magnanimity, all fear above;
From nobler recompense, above applause,
Which owes to man's short outlook all its charm."

### A CLEAR ISSUE.

It is highly desirable that everybody interested in nursing should realise the position in which the question of the registration of nurses is placed by the findings of the Parliamentary Committee. Casual observers, and even some who have taken a good deal of interest in recent controversies, have been inclined to think that the statement that the Parliamentary Committee in effect report against the scheme of nurse registration embodied in the two Bills submitted last session, in view of the fact that the same Committee agree that it is desirable that a register of nurses shall be kept by a central body appointed by the State, is rather a splitting of hairs. We are not surprised that such a view should be held by some who have not followed the controversy closely, and who do not apprehend the true inwardness of much which has happened in regard to this question of registration. First then it is necessary to realise the policy and aims of the two chief parties to the controversy. Many things have tended to obscure these issues which may be usefully re-stated at the present time.

On the one hand we have the authorities of the nurse-training schools, who have borne the heat and burden and cost of training nurses in this country from the outset. To these schools and teachers the British public are indebted for the trained nurses on whom the medical profession have to rely for the proper nursing of their patients. Some schools have taken the position that they have supplied most or all of the initiative, and that to them belongs the credit of providing the best system of training and the most valuable certificates which any nurse in this country can possess. They have held, and continue to hold, that they possess the right to make terms with their probationers when the training contracts are first entered into, whereby the nurse engages her services to the hospital and school for a period of four or five years, during which time her emoluments and allowances are fixed, and the whole of her services are placed at the disposal of the authorities. In this way, through the private nursing institution attached to some of the hospitals, a considerable revenue has been earned for the hospital by the nurses during the period of their training and contract. Those schools, however, which are entitled to claim that they best fulfil their duty to the public and the nurse, have so organised their private staff of nurses, that practically the whole of the surplus derived from the nurses' earnings, after paying expenses, is given back to the workers. Had the nurse-training schools been brought into co-operation, and had they adopted a uniform standard of training and contract, we should no doubt have been spared some controversy, and the higher education of nurses would have been in a much more satisfactory position to-day than it in fact is. It must not be forgotten, however, that the adequate training of nurses has been a progressive business, involving changes and expenditure which were not foreseen at the outset, and that it is only in recent years that the necessity and advantages of affiliating the nurse-training schools have made themselves felt.

In view of the confusion and diversity of system and practice caused by the circumstances we have just referred to, a few ardent and adventurous spirits in the nursing world have set themselves to force the position in what they conceive to be the best interests of the nursing body. These reformers have excited the attention of many ambitious people who, though they may have formerly been connected with nursing and the training of nurses, have retired from the business on their marriage or otherwise and ceased to hold any official position or to take any real part in this work for many years past. These ardent spirits, with their amateur backers, have opposed themselves to the authorities of the nurse-training schools, and by agitation, often carried to extremes, have striven to obtain for themselves a position which they have hoped to make sufficiently strong to induce the public and the nursing world to accept them seriously, and so to secure that Parliament should give them a predominant voice in the control of nursing education in this country, largely to the exclusion of the nurse-training schools.

It will be seen then that the controversy in reality has amounted to this. On the one hand the nurse-training schools have held that they are doing all that is necessary to promote the efficient training of nurses, and that their certificate is for all practical purposes adequate to protect the public, the profession and the nurse. On the other hand the reformers, with their amateur backers, have attacked the nurse-training schools, have striven to set the nurses against their authorities and teachers, and to get Parliament, and especially the House of Commons Committee, to find in favour of a Bill which would practically ignore the school authorities and place the control of nursing nominally in the hands of the whole body of nurses, but really in those of the ardent spirits and amateurs. After two years' exhaustive inquiry the Parliamentary Committee, by its findings, has rejected the case put forward against the nurse-training schools and has declared itself in favour of the co-operation of all nurse-training schools, and of the establishment of a register of nurses under a central body appointed by the State, which shall consist of matrons, nurses, representatives of the practitioners, of the training schools and of the public. In face of these facts we hope everybody will now appreciate at its true value the report of the Parliamentary Committee, which advocates the appointment of the General Nursing Council representative of all interests.

### Laryngeal Diphtheria.

By Mrs. HICKS, Formerly Matron of the Evelina Hospital for Sick Children.

### III. INTUBATION AND TRACHEOTOMY.

(*Continued from page* 71.)

Tracheotomy, which means incision of the trachea, is the alternative for relieving dyspnœa in laryngeal diphtheria.

This operation being performed in the ward, the nurse will have to prepare it accordingly. A high narrow table is covered with a double layer of blanket and sheet mackintosh; a stout short sandbag to be placed under the child's neck so as to throw out the part to be operated upon; a small mackintosh, two hot blankets, boxes with sterile coats, towels, swabs, gauze and wool will be required, and also plenty of warm lotio hydrarg. perchlor. 1-2,000, lotio carbol. 1-40, and lotio boric, as well as some sterile feathers.

The list of necessary instruments comprises : (1) Sharp scalpels, two pairs of dissecting forceps, four pairs of artery forceps, two small retractors, a director, a probe, one sharp hook, one pair of blunt-pointed and one pair of sharp-pointed scissors, one trachea dilator, one needle holder, sutures, and ligatures. (2) In a separate dish, the different tracheotomy tubes with their pilots or introducers.

The instruments are boiled in the usual way and placed in a dish containing lotio carbol. 1-40.

The tracheotomy tubes are boiled and then placed in a sterilized dish containing warm boric lotion.

Pilots with bone handles (which are only glued on) must not be boiled, but placed in lotio carbol. 1-20 for as long as possible, and then into boric lotion.

The anæsthetic table is prepared in the usual way—with the anæsthetics, inhalers, hypodermic solutions and syringes, tongue depressor, tongue forceps, gag, sponge holder and swabs, porringers, and towels.

Oxygen is frequently required in bad cases, and should be near at hand.

The most popular tracheotomy tubes are Parker's or Durham's, the old convenient bivalve tube having been discarded.

All classes of tubes vary in size according to the age of the patient. Each tube consists of three distinct parts—the outer tube, with its shield, the inner tube, and the introducer or pilot.

In Durham's tubes the inner tube consists of a succession of shell-like divisions (fitting into one another like a

Durham's (Lobster Tail) Tracheotomy Canula. (Inner Tube.)

lobster's tail), which will adapt themselves to the outer solid tube and make introduction very easy. The pilot is constructed in the same lobster-tail way, and the important point to remember is to introduce these tails into the outer tube by holding them in the direction in which the tail curves most. This is readily ascertained by holding it up, when it will be seen that it curves round very much more in one direction than in another, and only this greater curve will fit into the outer tube.

A great advantage of Durham's tube is that the length of tube passing into the trachea can be regulated to the depth required by a small screw in the shield, being careful to fix one tube perfectly straight. The shield is threaded on either side with a piece of tape, and it is best not to knot this, but to make a small incision near one end of the tape, pass the other end of the tape through the shield-eye,

Durham's (Lobster Tail) Pilot, with Outer Tube Affixed.

and then draw it through the slit previously made. By pulling it tight the tape will be in good position without causing any discomfort.

The pilot is only required at the time of the operation and whenever the tube has to be re-introduced. Be very careful to have each pilot tied to its tube when not in use.

At the time of operation the outer tube must be mounted on the pilot, the inner tube lying in the same basin.

If there should be any doubt as to which tube will be used, have each kind and size in question in a separate

Parker's Tracheotomy Tube.

bowl, because in critical times fatal delay may result from having the different tubes mixed up in one dish.

Parker's tubes are solid and unpliable; but the shield is movable; the tape is fixed in the shield in the same way as before mentioned. The inner tube is held in position by a small valve on the outer tube, which must be turned after the inner tube has been inserted. At the time of operation the

Introducer for Parker's Tracheotomy Tube.

outer tube is mounted on the pilot, the inner tube lying loose in the basin.

OPERATION.

The child is placed on its back, the back of the neck resting on the sandbag, the head well thrown back so as to project the part to be operated upon.

The order of the operation is as follows :—

| | *Instruments required.* |
|---|---|
| Incision ... ... ... ... | Scalpel, artery forceps, dissecting forceps. |
| Dissecting structures overlying trachea ... ... ... ... | Artery forceps, retractors, director, scissors, dissecting forceps. |
| Stopping hæmorrhage ... ... | Ligatures. |
| Pulling up trachea ... ... | Sharp hook. |
| Incising trachea ... ... ... | Dilator. |
| A few sutures may be required ... | Sutures and needle-holder. |

When the trachea is being incised be ready with the tubes, and also ready to catch any discharge coughed up through the incision. Hand a small dressing of dry gauze cut in this  manner, and tie the tapes round the neck tight enough to prevent the tube from slipping out. The knot should be at the side, not at the back, of the neck, for obvious reasons.

If the patient does not respond at once to the opening of the trachea artificial respiration is generally resorted to. It must be done slowly, with the patient's head low and the body raised. This position also assists the expulsion of any blood that may have found its way down the trachea during operation. Oxygen is as a rule administered as an additional help. These restorative measure must be persevered with for a considerable length of time. I have seen a patient recover who only started deep regular inspirations after artificial respiration had been performed for forty-five minutes.

Hypodermic injections of strychnine or other stimulants may be required, and let me point out here once more the necessity of having syringes and needles in perfect order.

Hot bottles and hot blankets are much needed during this period of exposure, and will be required for the cot as well.

(*To be concluded.*)

### The Nurses' Clinic.

### THE DISPENSARY. BY A CERTIFICATED DISPENSER.

### GENERAL HINTS.

Accuracy, neatness, cleanliness, and despatch are the qualifications most needed by a dispenser. All mortars, ugs, spatulas, and measure glasses should be left scrupulously clean, scale-pans wiped immediately after use, and all bottles and jars should be returned to their respective places as soon as finished with. "A place for everything and everything in its place" is a motto rigidly adhered to by a careful dispenser. A good plan is to have all bottles in alphabetical order on the shelves, but to keep all of a kind together; for instance, one shelf to contain all stock mixtures in their order, the next all oils, then spirits, tinctures, and so on round the room. Poisons should always be locked up in a cupboard. Acid hydrocyan. dil. should be kept in blue or amber-coloured bottles in a dark place. Lotions, liniments, or anything for external use only, should be sent out in coloured bottles, or bottles shaped differently from an ordinary medicine bottle, so that a person taking them up in the dark can tell at once by the feel whether they have hold of a medicine or lotion bottle, and may thus avert an accident. Care, too, must be taken over labels. Legibility of writing is of the utmost importance, and nothing must ever be left to the imagination of the patient; all of the doctor's directions must be stated fully and distinctly every time, no matter how often the patient has the same medicine; and the dose must be clearly and distinctly stated. Special labels as "shake the bottle," "poison," "for external use," should be placed near the shoulder of the bottle, that they may not be overlooked, with the directions for use underneath. Mixtures containing prussic acid (acid hydrocyan. dil.) must always have a "shake the bottle" label, as the acid being heavy sinks to the bottom, and if the bottle were not shaken the patient would take all the poison in the last dose with fatal effect. Such accidents have occurred. It is better to label all bottles for outward application with a "poison" label. A dispenser should err on the side of carefulness rather than run the slightest risk of causing an accident to a patient. Mixtures to be taken must never be labelled "poison," even if they do contain a small amount, unless specially ordered by a physician, otherwise a patient might think he was going to be poisoned and refuse to take the medicine. When in doubt as to a prescriber's intentions he should be seen or communicated with, if possible, but if that is impossible and the dispenser decides to alter the prescription, he should make a note to that effect for the guidance of others who may have to dispense it another time, and the prescriber should be told of the change made at the earliest opportunity. Dispensing can only really be learned by practice, no amount of theory will make a good dispenser without practical experience. Whole and undivided attention must be given to the work in hand; this applies more especially to this work than to almost any other, where a small slip, or a moment's distraction of thought may cost a life; the utmost care must be taken over every detail. A measure glass not properly rinsed may contain a small amount of poison, which used again without washing may be put into a draught and fatal results occur. Not long ago in Australia there was a case of poisoning caused by the dispenser having weighed out an ingredient on a scale-pan which he had omitted to wipe after having used

### THE NURSES' CLINIC—*Continued.*

for a poison, with the result that after taking the medicine the patient died. Numerous other instances might be quoted of the results of carelessness on the part of a dispenser. No one should take up this work without first considering the grave responsibility it entails, and the fact that many lives are in their hands, and of their own fitness for handling what has been beautifully described as "the mysteries of God."

The first thing to do on seeing a prescription is to read it carefully through, noting any unusual doses or incompatibilities; if there are several prescriptions to be done, it is always best to do the easiest and shortest ones first, leaving the more difficult ones until the end, when more time can be given to them knowing they are the last, than with the feeling that there are several others to be done. If an unusually large dose be ordered it is best to consult the prescriber, who in the case of a poisonous overdose should always put his initials against it. Some poisons can, by beginning with small doses, be gradually increased until the patient is taking quite a large one.

The doses in the British Pharmacopœia are not unalterable, and often considerably more than the official dose can be given. Prescriptions should be dispensed as quickly as possible, slow dispensing is bad dispensing; poisons should always be added last, to lessen the risk of their being put in twice. It is best, if possible, to keep a special measure-glass and mortar for poisons only. Another special glass should always be kept for measuring oils in, the simplest way of cleaning which will be found by using sawdust. In dispensaries, where space is limited and there are many bottles, it is a good plan to keep some mixtures, as M. Cretæ, B.P., and M. Guaiaci, B.P., with all the ingredients ready mixed in powder form, adding the Aqua Cinnamomi when required. Thus a great deal more can be kept in stock with little or no extra trouble. Most soluble salts are kept in solution. The following is a list of the most usual ones:—Acid citric, soluble, 1 in 2 of water; Acid boracic, soluble, 1 in 16 of water; Amon. carb., soluble, 1 in 8 of water; Magnesii sulphas, soluble, 1 in 2 of hot water; Potas. bicarb., soluble, 1 in 4 of cold water; Potas. bromid., soluble, 1 in 4 of cold water; Potas. chlor., soluble, 1 in 16 of hot water; Potas. iodid., soluble, 1 in 2 of cold water; Potas. nitras, soluble, 1 in 4 of cold water; Quin. sulph., soluble, 1 in 4 of acid sulph. dil.; Sodii barcarb., soluble, 1 in 12 of water; Sodii salicylas, soluble, 1 in 2 of water.

Therefore, in a prescription with, for instance, one scruple of ammon. carb. in the whole mixture eight times that amount of the solution should be put in. Or, again, we will take an imaginary prescription:—

| | | |
|---|---|---|
| ℞ | Ammon. Carb. ... ... ... ... | gr. xl. |
| | Sodii Bicarb. ... ... ... ... | gr. xl. |
| | Tr. Scillæ ... ... ... ... | ʒi. |
| | Tr. Camph. Co. ... ... ... ... | ʒi. |
| | Aqua ad. ... ... ... ... ... | ℥viii. |

It will be seen that five drachms twenty minims of the ammon. carb. solution—that is, eight times more of solution than if the powder were weighed—and one ounce of sodii bicarb. solution—that is, twelve times 40 grains—will be wanted. With water 'ad' (up to) eight ounces. Thus it will be seen that a great deal of time will be saved by using solutions of salts instead of stopping to weigh them out on the scales; and with a little practice the calculations become very simple. Some doctors write their prescriptions for the whole amount required to be sent; others, again, writing theirs for the dose only, directing how many are to be sent. Care must be taken to read the whole prescription through before commencing to compound it, otherwise after having made up a mixture to an ounce or so the careless dispenser may discover at the end 'send six ounces' or eight as the case may be, and the whole thing has to be done over again. The signs representing ounces, drachms, etc., should be learnt before attempting to commence dispensing. They are very simple: ℥ meaning oz., ʒ = 1 drachm (dr.), ℈ = scruple (sc.), m. stands for minim, and gr. for grain. Half is represented by ss., thus ℈ss. means half a scruple; ʒiss. stands for one drachm and a-half. The apothecaries' and avoirdupois tables must also be committed to memory; and it is most important to remember that a minim is not the same weight as a grain: there are 480 grains in an ounce, but only 437½ minims to a fluid ounce. A dispenser must get into the way of calculating rapidly, and correctly. In most prescriptions there is a certain amount required; and lotions are often ordered, for eight or six ounces as the case may be, at 3¼ or 2½ per cent. After a little practice it comes very easily even to those who are not good at arithmetic. A certain amount of knowledge of Latin is essential, as is a knowledge of the abbreviation of Latin words. Everybody knows the story of the chemist's assistant who had a prescription to make up with the words *Pro re nata* in the directions; not knowing exactly what it mean the looked up *nata* in the dictionary, and, finding it was to do with "birth," he labelled the bottle "for the baby," never having learnt that it was a phrase meaning "occasionally" or "according to circumstances"!

### Incidents in a Nurse's Life.

Contributions to this column are invited.

### A CASE OF EMBOLISM IN A COTTAGE HOSPITAL.

There were two women and a child in the hospital, no men patients. The women were, a subacute appendicitis, a chronic ulcer of ankle, and the child a tubercular knee: the staff, one nurse-matron, one servant.

It was about the end of August when the doctor brought in a big man with a mild attack of typhoid. He was in the second week, not really very ill, but he lived in a very small cottage without much air, so that it was deemed wiser he should come into hospital. The temperature was never over 102°, and the patient felt the effects of the fever so little, that he hardly thought it worth while staying in bed. He was the 101st case of typhoid which I had nursed and seemed likely to be one of the mildest. The illness had been traced to the man having eaten about four raw cockles while on his round as a miller's drayman. The first fourteen days were uneventful; the temperature had come down to normal, then it gently rose, and he had a mild relapse, lasting a week, but on the eighth day it had again reached normal. On the ninth day, being Sunday, it seemed possible for me to go to church, but, finding that the temperature was again 100°, church was once more given up; one of the drawbacks of working single-handed. I questioned him as to any fresh symptom. He only complained of a pain in the right thigh. He put his fingers a little roughly on the spot, and I told him he must not touch it for any reason. While I still stood by his side he coughed, and shook himself a bit, and, with a cry, fell back, with blanched face and groans, saying he was being cut open, tracing a pain from his hip to the chest. I rubbed brandy on his lips, ran downstairs to send the servant for the doctor, and returned to him. He was almost dying, cold, clammy, blue. How helpless I felt! The doctor came quickly and said he had a large clot in the heart and would most likely die. He said that it was a wonder he had not died at once. He gave me some pure ether to inject if he got worse, as he was going into the country, and I must trust the rest to Providence. His wife was sent for and his danger explained to her. She stopped all that day, and only left after ten at night to go to see to her children. I remained with him all night, expecting his death almost hourly. He was given ether, brandy, and sal-volatile at short intervals, and in this cold, clammy grey condition he remained all the night. Then the doctor thought he might live three days. He came four times during each day, and there was nothing to be done but wait. He was gently sponged with a very soft sponge, but no towel was used for fear of the result of friction. The clot slowly contracted, the temperature having shot up to 105°, remained so most of the time. The pulse was feeble and thready. On the following Sunday the doctor missed his fourth visit, but after all I had to send for him. He merely thought that I was nervous—I had not been to bed yet—but he came and found that the clot had moved from the heart into the right lung. The symptoms were much the same, but less urgent. The illness now developed into a bad case of pneumonia, the whole lung being quickly involved. Jacket poultices were ordered and kept on for the next eight days. I invented a way of my own to put them on, one side at a time, instead of back and front. The heart being very weak, brandy was increased, and at last reached 10 oz. per day. Ether, brandy, milk, and raw meat juice were given every twenty minutes, and the patient's feeds worked out at seventeen a day. His wife spent all the time she could by him, and while I did my other work, and went out shopping, she was there. I used to put the feeds in glasses marked with the times due, and she gave them to him.

On the fifteenth day I was spent. I could no longer understand figures, such as the pulse and temperature. I had not yet been to bed, so a nurse came, who was on her holiday, and I went to bed.

For five weeks the pneumonia ran its course, and ended in fluid forming. Twice aspiration was performed, and four quarts of pus removed. After that came hæmorrhage from the lung; then a period of delirium, and in imagination he drove his horses and carried his pillows like sacks of flour. The cough was the loudest and longest that I had ever heard. It is difficult to realise that a patient could be so dangerously ill for so long, but the whole of those seven weeks his life hung on a thread. At last gradually the pulse improved. I could once more do without a night nurse, and convalescence appeared in sight. He was in the hospital altogether for eighteen weeks, and at last went out, quite well. He almost wept when he left, and I always feel proud when I meet him, strong and hearty, driving the horses he loved so well.

### A Business Trip to the United States.

BY A PRIVATE NURSE.

I was nursing in the South of France and had just returned to the Institute when I was told that a nurse was wanted to take a patient "slightly mental" to America. The most important point was that she must be a good sailor. Now, it had always been my ambition to go to America, and as I had done five voyages between India and England, I thought that I was well seasoned. so I offered my services. Next morning the patient's father arrived at the Institute, and I was sent to him to be inspected. He was a 'cute American and was worth many dollars. He looked me up and down as if I had been a bale of cotton, asked in a few questions, "Do you think you could manage a case of mental trouble?" etc. Then said, "D'you want to go to Amurrica?" to which I answered that I had long wanted to go there and that I was a good sailor. "Wal, if you go and get sick I'll just throw you overboard." I promised not to be sick without great provocation, but could not guarantee anything. Then he said, "Will you come? Right! I'll be here in my automobile in two hours; your trunk can follow by rail." As he was going down the steps he turned round once more with "What's your name? Gertrude; wal, none of your miss or nurse for me, I'll call you Gertrude." And he did to the end. He was a quaint person in many ways. In less than two hours he returned with my patient, and my heart sank when I saw her, for she was more than "slightly" mental. The motor was a beauty and we did twenty-five miles up hill to Grasse (where we were to stay for a fortnight before going to America) in a little more than an hour: the scenery was grand and the dust was dreadful; we were all white by the time the journey was at an end. The next fortnight was a very anxious time for us all. The patient was much worse than she had ever been and we wondered if we should get her to New York alive. I had to ask her mother to engage another nurse for the voyage, as she needed to be watched day and night and I was about tired out and could not undertake the journey alone. Our hotel was just at the side of a hill looking out over Grasse; the view was glorious from our balcony, two sides were hills, and away to the south we looked over Cannes, on to the Mediterranean. Grasse is a pretty old town, with straggling streets, and very hilly; the Cathedral is perched away above the rest of the buildings. There were many scent factories, but the perfumes did not always remind one of Araby, and the breezes were not always spicy in the narrow streets, for sanitary arrangements were primitive. The weather was perfect, and I enjoyed my very short hours off duty, explored the old place to my heart's content, and lost myself many times in the "slums." The peasants had rarely seen "une garde malade Anglaise" and I was an object of curiosity as I went by; they used to make remarks in a loud and cheerful manner, but I got accustomed to that and would say "Bon jour, madame," just to show that I could understand French. We had a trying journey to Genoa (the other nurse joined us at Nice, to my great relief) and spent the night there. We could not see much of the town, but Christopher Columbus, carved in marble, lived in the square opposite, so we had a good view of him. Next morning we sailed in one of the Nord Deutscher Lloyd boats, a floating hotel, very large and luxurious. Our patient improved wonderfully after we had been at sea a short time, and the voyage really did her a great deal of good. On Friday we were at Naples; its harbour is beautiful, and Vesuvius was smoking gaily in the distance. I went on shore with the parents of my patient. They wanted to buy gloves and tortoise-shell combs. We lunched at the Metropole, looked round a few shops, and returned to our boat. On Monday we arrived at Gibraltar. It was a fine day and we went on shore, drove all round the old rock, saw the docks and fortifications. My American friend said that I "put on side, and looked no end proud because I was on British territory." Well, I confess I was a little proud to think that it all belonged to our "tight little island," and the English Tommies were so smart after the French gendarmes. We did not stop at any more ports until we arrived at New York. We passed the Azores early one morning in the sunrise; they were beautiful, so green and fresh, with little pink houses nestling in

A BUSINESS TRIP TO THE UNITED STATES—*continued.*

under the hills. They looked thinly populated and not very gay. People talk of the Bay of Biscay; let them try the Gulf Stream for a change—what a time we had! For four days most of the passengers were laid low and the saloons were very empty at meal times. I was sick once, but the provocation was great and I did not report the fact until we were on terra firma once more, as I did not feel ambitious of feeding the monsters of the deep with my anatomy. A few days later we arrived at New York in a snow-storm, and after a little delay drove to our hotel, a great building about twenty-four stories high. We remained there a night. Next day our patient and her parents went to Boston to a sanatorium. Nurse and I were left in New York by ourselves. Rooms had been taken for us at a boarding-house, where we were very comfortable and enjoyed the experience. Our fellow boarders were friendly and kind, and as nurse had a few friends there we had our time well taken up. An English nurse in uniform was a rare object in that part of the world, for the nurses in America do not wear outdoor uniform; people took quite an interest in us and used to ask if we had come from the "other side," also what we thought of "Amurrica" and "N'York." The weather improved and we had "a real good time"; we were quite sorry when we had to leave for Genoa. Another tossing was in store for us (it was worse than the first), in the Gulf Stream, where we had five days' dreadful weather. I was sick many times and very glad that my American friend was not on board. Upon our arrival at Genoa we dined on shore at a café, and after dinner drove all over the town, returning to the steamer about 11 P.M.; the captain allowed us to sleep on board and so saved us the trouble and expense of going to the hotel for the night. In the morning we went on shore again, looked at the shops, visited two or three churches, invested in more postcards, and then made our way to the railway station in time to catch our train for Nice Thus that experience was ended, much to my regret, for it had been very pleasant and one on which I shall always look back with happiness.

### Two Little Charges: Motherless Twins.

By the untimely death of a young mother the care of twins less than a month old fell into my hands. Had the doctors not declared that their existence was at the most only a matter of a few weeks, I should scarcely have had the courage to undertake the charge, as my hospital training—owing to health and other causes—had been of somewhat a fragmentary nature, although the experience I had gained in 10 or 12 years' work had been rather wide.

### Left to Myself.

The family doctor called the day after my arrival and said that unless I sent for him he should not come for a week as there was really nothing that he could do. As I had only an ordinary children's nurse, the monthly nurse having been suddenly called to take another case, I felt the responsibility very great. When I took charge the babies were three weeks old and weighed only six pounds each, nearly one pound lighter than when they were born. Milk and water in its ordinary state had been tried for them, also several patent foods, but all had more or less disagreed with them. They looked so frail that it seemed a very hopeless task to try and rear them, but as their family and the doctor left them entirely in my hands I felt I could but experiment and hope for the best. It was one of those cases which no doubt occur in the life of every nurse when the ordinary treatment one has been accustomed to in hospital seems to help so little, and one has to rely on one's own judgment and meet the emergencies day by day as they arise, and just feel thankful when each day and each night is safely passed. For the first week particularly, and for some little time after, I simply had no rules either for feeding or sleeping, nor could I see much progress excepting that they lived.

### Still Alive.

When the doctor came as promised at the end of the week, the only remark he made was, "Well, nurse, they are alive, and that's something." I began by peptonising the milk, adding two-thirds water, and to each pint of fluid I added one tablespoonful of thick cream and a little sugar of milk. This agreed with them; but they only took a very small quantity at a time, not more than one ounce, and that was very often from a spoon, as they were too weak to suck frequently from a bottle. I do not think they were ever over one hour without food at this time, either day or night, and I kept them in one room with an equal temperature, but plenty of ventilation. Besides their binders and shirts, they wore only loose flannel gowns, as they were too frail to dress in ordinary clothes; but I sponged them in warm water all over morning and evening. Excepting for an occasional few minutes' superintendence of the household (there were four other children) during this anxious time I never left them, and the nurse was very good in getting up during the night when I could not manage them alone.

### First Improvement.

At the end of the first fortnight there was a decided improvement, they had both gained in weight, awoke less frequently and took a larger quantity of food at regular times. I now began to treat them as ordinary babies, peptonising their food less, but still adding the cream, until they were eight weeks old, when I left off peptonising entirely, but, for their 6 P.M. feed, after their bath, I gave each of them a bottle of Mellin's food. I always laid each one in his cot for this feed, and they dropped off to sleep without any trouble. I never gave them teats to suck, and of course I used the feeding-bottles without tubes. At three months old they were vaccinated with good results, and on the day they were seven months old they each cut their first tooth. Until the babies were a year old I took charge of them entirely at night, as I felt I could not expect nurse to do justice to them by day unless she had undisturbed rest, and, as during the day I had other demands on my time, it was impossible for me to be with them continually.

### Mapping out the Day.

My plan for the day was as follows:—The babies usually awoke between 5 and 6 A.M. I gave them each a feed, and at 6.30 nurse came for one and took him to the nursery to bath and dress him; this took her something less than half an hour. She then brought him back to me, taking the other one for the same duties. The newly-dressed babe was quite good, and amused himself either on the bed or the floor whilst I dressed. I then went with him to the nursery, and took care of both of them to allow nurse to go to the kitchen for her own breakfast. When they were old enough to have breakfast, such as bread and milk, or bread crumbled and covered with the drip from the bacon, and a cup of milk and water, nurse brought this up with her when she came from her own breakfast. I then left her to feed them, whilst I had breakfast with the rest of the family at 8.15. At 9.30 I returned to the nursery and helped nurse to dress the babies for their morning outing, as, if possible, I

always sent them out twice in the day. They generally had a little milk before starting, and at 12 they had dinner, after which they were put to bed for two hours. During the winter they could not always be taken out in the afternoon, so I used to give them a change of room and, after wrapping them up, open the window as much as possible. They early acquired the habit of lying on the floor, and I may say that after the age of two months they were practically never nursed, and this, I think, is one of the chief reasons why they grew so healthy and were so even tempered. One child was much troubled with constipation until he was a year old; after that period, with, I suppose, the more varied diet, and being able to run about, the trouble disappeared. I left them much to my sorrow, when they were 18 months old. They could then walk well, and were in the best of health. They are now nine years old.

The Nurses' Lessons to be Learnt.

The chief lessons, I think, one learns from a case like this are: First, that so long as an infant breathes there is a chance of rearing it. Second, the value of feeding in small quantities and frequently, irrespective of rules, until the child's individual capacity and powers of digestion are fully understood. Third, the anticipation of every need, so that no strength shall be lost by fretfulness. Fourth, the disadvantage of perpetually having a child in one's arms. Fifth, the benefit of two baths daily; and, lastly, the firm belief that children are sent into the world to live.

### Practical Hints.

We welcome notes on practical points from nurses.

### A FEW HINTS ABOUT LEMONS. BY A SISTER.

That the homely lemon should be so popular with the sick is not surprising when one knows how it can be utilised. The yearly import of lemons is so considerable that it in itself is irrefutable evidence of the demand for them.

First, as a mouth cleanser. Every good nurse knows how important the care of the patient's mouth is, especially in cases of pneumonia or typhoid, and, indeed, in any illness where the temperature is high and when the diet is limited almost entirely to fluids. To make an excellent and refreshing mouth wash, add the juice of a lemon to about two teaspoonfuls of glycerine, mix well and apply with lint or linen rag attached to forceps. If the patient be strong enough to use a mouth-wash himself, an ounce of hot water may be added to a teaspoonful of the mixture, this will prove pleasant and efficacious cleanser.

Often after an anæsthetic patients complain of a feeling of sickness and a "nasty taste" in the mouth. To relieve the sufferer, a lemon may be thinly sliced and cut into small pieces which the patient may be allowed to suck. These small sections will also prove most acceptable when "nasty" medicine has to be taken—more especially, perhaps, the unfortunate recipient of castor-oil. Let the patient squeeze a little juice into the mouth first, then add some of the emon juice to the dose of oil. These precautions will make the dose much less unpleasant to take and much less likely to produce vomiting.

Then, again, it is impossible to over-rate the value of the lemon in the making of drinks for patients. To make a really nice lemon drink, pare the fruit very thinly, take off the white coating, then slice the lemon and add some of the yellow rind and a few lumps of sugar, if desired, pour on a pint of boiling water, cool down, and ice if hot weather. Some patients like barley water flavoured with lemon juice; this is both nourishing and refreshing. Another nice variety may be made by adding the juice of a lemon to a tumbler of soda-water. Custards, blancmanges, junkets, and other puddings of the milky tribe so useful in farinaceous diet, may often be made more palatable and appear more varied to a patient if flavoured sometimes with lemon, at other times with almond or vanilla essence.

Now as to the value of lemons to the nurse herself. If owing to chilly East winds or to the use of hard water the skin should become rough an excellent emollient is the juice of a lemon added to an equal quantity of glycerine and rosewater. This applied as often as possible after washing one's hands and always on retiring for the night, proves very softening and keeps the skin in good condition.

For nurses on their holiday a lemon sliced and put into a gallon of cold water makes a refreshing wash for the face after exposure to the sun or wind. If the feet are tender and tired in hot weather, besides the usual ablutions and changes of stockings and shoes, lemon juice well rubbed into the soles of the feet will make them much less tired and relieve the soreness. Some maintain that a compress of lint soaked in lemon juice and applied to corns and bunions is good; it certainly relieves the pain, and no doubt, in some subjects, is curative. Anyhow, it is worth a trial, if only for the relief occasioned.

A tumbler of hot water, with a little lemon juice squeezed into it, is a very good thing to take before breakfast, and if some people who suffer from indigestion and constipation would try this instead of the more usual cup of tea they would probably suffer less from these complaints.

For a headache which accompanies a bilious attack, lemon juice in a cup of fairly strong but freshly-made tea is excellent, and often gives relief. It also makes an agreeable addition to the after-dinner cup of coffee, assisting in the counter-action of the too rich dishes and their attendant evils. From a strictly utilitarian point of view the lemon has excellent qualities. To remove stains from the fingers after paring fruit or vegetables rub with the rind of a lemon. Straw hats of the plain white kind may be cleaned beautifully by applying lemon juice with a brush. Ink stains, too, when fresh, may be removed from white linen by soaking the affected part in a mixture of lemon juice and salt for an hour or two then washing with soapy water. This is a useful hint, for nurses sometimes have the misfortune to get a spot of ink on the proverbially spotless apron or uniform; a real disaster to a nurse who prides herself on her trim appearance.

### To Nurses.

We invite contributions from any of our readers, and shall be glad to pay for "Notes on News from the Nursing World," or for articles describing nursing experiences at home or abroad dealing with any nursing question from an original point of view, according to length. The minimum payment is 5s. Contributions on topical subjects are specially welcome. Notices of appointments, letters, entertainments, presentations, and deaths are not paid for, but we are always glad to receive them. All rejected manuscripts are returned in due course, and all payments for manuscripts used are made as early as possible after the beginning of each quarter.

### New Books for Nurses.

Medical Electricity and Light Treatment. By Kate Neale, Sister in Charge of the Actino-therapeutic Department, Guy's Hospital. (London: The Scientific Press, Ltd., 28 and 29 Southampton Street, Strand, W.C., 1905. Pp. 98. 20 illustrations. Price 2s. 6d. net.)

All light treatment ought, of course, to be illuminating. However true that may be, it is equally so that some treatises on the subject are the reverse, and serve only to bewilder the would-be student. This cannot be said of the little handbook now before us, which, by reason of its perspicuity, should be most useful to nurses interested in this branch of modern treatment, or to those passing through that department as part of their hospital training. It is couched in as simple language as is compatible with the unavoidable technicalities of science, and these are explained as soon as met with. The whole subject naturally being approached from the nurse's standpoint, instead of that of the medical man or the scientist, is therefore much more comprehensible to those who have not made a special study of electricity and magnetism. Combined with actual experience and practical demonstrations of the various workings of the various apparatus mentioned, the handbook should carry a nurse successfully through her work, and help to make her an intelligent coadjutor in the doctor's treatment of his patient. The chapters on the Finsen light are very interesting, and much information is condensed in less than 20 pages devoted to this subject. The many dangers attendant upon the methods of treatment dealt with are all emphasised in such a way that burns and other injuries are only likely to occur through carelessness, and not ignorance, on the part of the nurse applying them, if she has studied as she should the clear directions given for avoiding such unfortunate accidents. The type, paper, and general get-up of this book are very good, and the illustrations are convincing in their accuracy. The nurses at Guy's are to be congratulated on their teacher, as well as on their admirably fitted up department, where they may learn something of such deeply interesting and useful methods of treatment.

Nursing: Hints to Probationers on Practical Work. By Mary H. Annesley Voysey. (London: Scientific Press, Ltd., 28 and 29 Southampton Street, Strand, W.C. 1905. Pp. 111. Eleven diagrams. Price 2s. net.)

For presentation to the intending candidate, or as a gift to the probationer in her first year of hospital life, this little book will no doubt be acceptable and useful. Its style is simple, direct, and practical, its English homely and unpretentious, and it does not aspire to deal with matters that are too involved. On the contrary, simple details of ward work are treated with a thoroughness that leaves nothing to be desired, showing a wide and comprehensive knowledge of the weak points of probationers, and it should save the tyro in hospital work from making many awkward mistakes during those fearsome early days of her probation. Some clear diagrams, together with a good index, and the heavy lettering which marks the commencement of a fresh subject, makes the book an easy one for reference, while the true nursing spirit of devotion and care for the comfort of the patient pervades it all through, stamping it as an earnest and helpful little work.

Simple Sanitation. By M. Loane and Dr. A. M. Fraser. (London: The Scientific Press, Ltd. 1905. Pp. 80. Price 1s.)

This book recommends itself by its simplicity and clearness in detailing many of the more elementary, though none the less important, matters connected with sanitation and hygiene. Besides setting down the householder's responsibilities with regard to water, air and ventilation, drainage and disposal of sewage, two chapters are devoted to household management and personal hygiene. Busy district nurses and midwives will find a great deal of valuable and practical information in small compass, and they will be enabled to recognise, minimise, and avert dangers to which their patients may be exposed. It is to be hoped that the teaching in the book will become generally known, because if acted on much will be done in the home to prevent sickness and disease.

The Apsley Cookery Book. By Mrs. J. Webster and Mrs. F. Jessop. (London: J. and A. Churchill. 3s. 6d.)

This book contains 448 recipes for the uric acid free diet. The recipes are vegetarian, and of a limited description, as a glance at the specimen menus shows. For instance, breakfast may consist of—milk, toast and scones, fried potatoes, and nuts. Lunch—rice (savoury), mashed potatoes, bread-and-butter pudding, ground rice mould; and dinner—purée blanc, curried vegetable and rice, cauliflower fritters, and potatoes. For those who intend to adopt such a diet as is indicated the book will form a valuable guide, and it will greatly help a nurse who has charge of a patient who is obliged to do likewise. The value of the book is enhanced by a table of food values compiled from Attwater's Tables.

The Model Kitchen. By Lucy H. Yates. (London: Longmans, Green, and Company. Price 2s. net.)

In "The Model Kitchen," Miss Yates tells us how to fit up, arrange, and utilise a kitchen to the best advantage. Her work is a cookery-book in the fullest sense, but it is not a collection of heterogeneous recipes, gathered from all manner of sources by a compiler who knows nothing personally of the subject which describes the majority of cookery books. "The Model Kitchen" should act as an inspiration to the inexperienced young housewife, who from lack of knowledge and interest is so often at the mercy of an ignorant and unskilled cook. In reading Miss Yates' book she will see that cookery properly understood is a delightful art, which in reality should hold its place amongst the sciences, seeing the important part it plays in relation to health. "The Model Kitchen" shows us that the results of cooking in a certain manner, and not in another, are definite. The rules laid down are the outcome of experience and knowledge. It is a pity that there is no work extant, as far as we know, which explains the scientific phenomena which take place in process of cooking. A work like "The Model Kitchen" awakens a taste for investigation.

### Novelties for Nurses.

(By Our Shopping Correspondent.)

### CORSETS FOR INVALIDS.

Messrs. Debenham & Freebody, Wigmore Street, London, W.

The Maillot corset is one that can be safely recommended for invalids and for those recovering from operations. It not only combines elegant appearance with comfort, but keeps the figure in place without any ill effects. An important feature of this corset is that it is made entirely without bones, a fact which renders it peculiarly adaptable to the requirements of invalids and convalescents. If desired, bones can be inserted to suit the wearer. Being made of woven silk it also provides ample warmth. The Maillot is not so high as the ordinary corset, but a small silk corslet can be obtained if required. Madam Zilva, who has had much experience in this special branch, personally supervises all the details of the construction, so that complete satisfaction in every way is assured.

### A Tragic Moment.

It was the dinner-hour at the workhouse infirmary, and a young nurse was alone on duty in the infirm wards. These wards were on the top floor of the block, and possessed a large sunny balcony, where the old men basked in the sunshine on fine mornings. Sister was quietly scanning a newspaper, waiting till the big clock pointed to half-past one; she often joined the first batch of nurses for dinner, but from some mysterious impulse to-day she decided to join the second batch. It only wanted a few minutes to the half-hour when down rushed a breathless messenger, with the startling cry, "Oh, sister, will you come up; a patient is choking—one of the 'daddies' in the infirm ward?" Away flew sister, and in a few seconds was at the top of the stairs, and upon the scene of the threatened tragedy. Rapidly rushed through her brain the impossibility of securing any efficient help. Matron was at dinner, and the doctor had probably gone to his lunch, for he lived about a mile away from the infirmary. But she resolved to act promptly, and, if possible, save the patient. In a small ward lay the old daddy, flat upon his back. Suffocation was imminent, as evidently respiration was suspended, and the livid purple face showed every sign of asphyxiation. Round the bed stood a few horror-stricken patients, and the young nurse perfectly helpless with terror. Without a moment's hesitation sister raised the patient to a sitting posture, inserted her finger into his mouth, and gradually worked her way to the back of the object that was obstructing the patient's breathing. With a great effort she succeeded in dislodging what proved to be a thick, solid piece of meat, which the old man had despatched without any attempt at mastication. Breathlessly her white-faced junior watched the proceedings, and great throbs of intense relief filled that young damsel's breast as she watched the natural colour returning to the old daddy's face, and knew that a tragedy had been averted which she herself had been powerless to prevent.

After calmly giving a few necessary orders, and dispersing the frightened patients, sister explained the situation to matron, who had arrived too late to render any assistance, and went away to her dinner, a trifle paler than usual, but otherwise as serene as though choking-patients and life-saving were quite an every-day occurrence.

### Appointments.

Brighton Workhouse Infirmary.—Miss Jane Mackay and Miss Florence Morton have been appointed staff nurses. Miss Mackay has been charge nurse at Portsmouth Parish Infirmary. She has also been nurse at a medical surgical home at Southsea, and at Woodbridge Hospital, Guildford. Miss Morton has been assistant nurse at Battle Union Workhouse and nurse at Ticehurst Isolation Hospital.

City Hospital for Infectious Diseases, Newcastle-on-Tyne.—Miss Edith A. Addison has been appointed night superintendent. She was trained at Westminster Hospital, London, and has since been nurse at the Fountain Fever Hospital, Tooting, London, and sister at the West Ham Infirmary.

Highfield Infirmary, Liverpool.—Miss Annie B. Dane has been appointed assistant matron. She was trained at the Western Infirmary, Glasgow, and has since been assistant superintendent at the Fever Hospital, Cork Street, Dublin, and night superintendent at the Royal Victoria Hospital, Belfast.

London Homœopathic Hospital.—Miss Victoria Daunt has been appointed lady superintendent. She was trained at the Great Northern Central Hospital, London, where she was afterwards sister. She has since been night sister, housekeeper sister, and acting matron of the National Hospital for the Paralysed and Epileptic, Bloomsbury.

Mold Cottage Hospital.—Miss Ida T. Gillett has been appointed nurse matron. She was trained at Croydon General Hospital and has since been sister at the Victoria Hospital, Blackpool.

Newport County Hospital.—Miss May Whitburn has been appointed staff nurse. She was trained at Sheffield Royal Infirmary, and has since done private nursing in connection with Newport County Hospital.

North Shields Jubilee Hospital.—Miss Emelie Ault has been appointed charge nurse. She was trained at Clayton Hospital, Wakefield, and has been nurse at the North Riding Asylum, Yorkshire.

Wharfedale Union Joint Isolation Hospital.—Miss Latchford has been appointed charge nurse. She was trained at the Nottingham Union Infirmary and the City Hospital, Liverpool, and has since been sister at the Fulham Infirmary.

Wimbledon Cottage Hospital.—Miss A. M. Cordner has been appointed staff nurse. She was trained at the City of London Hospital, Victoria Park, E., and Mill Road Infirmary, Liverpool, and has since been attached to the Victoria Nursing Home, Harrogate.

### Everybody's Opinion.

[Correspondence on all subjects is invited, but we cannot in any way be responsible for the opinions expressed by our correspondents. No communication can be entertained if the name and address of the correspondent are not given as a guarantee of good faith, but not necessarily for publication. All correspondents should write on one side of the paper only.]

### THE CENTRAL MIDWIVES BOARD AND ASTON UNION INFIRMARY.

The Matron of Aston Union Infirmary writes: There is no consternation at Aston Workhouse Infirmary. But we certainly do not consider that we have been treated fairly by the Central Midwives Board, because their inspector was told that the Guardians would make any alterations she considered necessary, and she left us with the impression that she was fairly well satisfied. Even if we do not become recognised as a training school for midwives, our probationers have the advantage over infirmaries and hospitals, where there are no maternity cases, as they attend the cases and have three months' excellent training in monthly nursing, for which they will be examined by an independent examiner and have the opportunity of gaining a certificate. Notwithstanding "that the structure and general conditions of the Infirmary" are considered unsatisfactory, during the last six years, with 430 births, not one mother has died in childbirth, and we have not had a single case of puerperal septicæmia. Can any institution show a better record? It is at least open to question whether midwives who have been trained where there are elaborate appliances will be any more competent to act as private and district midwives and know how to make the best of ordinary conditions.

### IRISH CERTIFICATES.

"A Dublin Hospital Nurse" writes: I understand that the authorities for State registration will require nurses to have certificates for three years' hospital training before they can be registered. Does this mean that they must have three years' consecutive training in one hospital, or, if a nurse has a certificate for two years' training, and in addition can get another year's training in the same hospital (and a certificate to that effect), or, failing that, an extra year in any other recognised training school, will she then be qualified for State registration? Most hospitals in Ireland train their nurses for only two years in the wards, and, no matter how many years we may afterwards serve our respective hospitals, either in hospital or on the private nursing staff, we get certificates for only "two years' training" at the end of our terms of service. Consequently, when we apply for appointments afterwards at other institutions, either in Ireland or elsewhere, we find ourselves disqualified by reason of our certificates. Take, for example, the "services," each of which requires candidates to have certificates for three years' consecutive training in the wards. Again, 99 per cent. of the advertisements of posts for trained nurses insist that the candidates "must have had three years' training." Will the superintendents or committees who advertise these posts (I presume they are the judges in this case) accept as eligible for the appointments nurses who have obtained their training at intervals, or do they consider the consecutive training indispensable? What, then, is to become of nurses who have only two years' certificates? . . . Shall we be disqualified for State registration? These are questions that Irish nurses are most anxious should be clearly understood and answered once and for all. We feel that we are labouring under great disadvantages, and we cannot understand why we should be deprived of what we justly consider as our right—*i.e.*, to have certificates which will place us on an equality with the nurses of England and Scotland, which at present we certainly are not. . . .

[There is no State registration at present, and whenever the subject is seriously considered by Parliament, the question of the nurses on whose behalf you write would certainly demand attention.—Ed. The Hospital.]

### DISTRICT NURSES AND MATERNITY WORK.

"One Interested" writes: If "Puzzled" were to have the good fortune to be appointed superintendent of a county nursing association she would be able to look on the combination of general nursing and maternity nursing in a less narrow-minded light. Although I quite realise the ideal theory of keeping the two works entirely separate in large towns, and even small towns, where it is possible and quite easy, I would bid "Puzzled" remember that all over England there are to be found villages of 200, 100, 50, and less inhabitants, and also miles and miles of country with only a few houses scattered here and there. Are the few poor suffering souls amongst them to be left to the mercy of a kind but ignorant neighbour because the district nurse, whose salary between them they manage with difficulty to collect, may only attend general cases or maternity cases, as the rule of those in authority may be? I know of many a district in itself made up of four or five villages, where the inhabitants with difficulty manage to collect sufficient to keep one nurse. Perhaps "Puzzled" does not realise that the nurses who do this work are trained in the law of antiseptics, and so taught to avoid risking their patients' well-being. Perhaps, too, she does not know that the maternity case always takes the prior claim, and if a doubtful case is on the books when a maternity case "comes off," the friends of the former are instructed, having probably watched the nurse many times previously, how to attend to their patients during the nurse's enforced absence from that house. If those who so repeatedly denounce the practice of combining the two works would give of their substance in order to provide for two nurses in the country districts where the two branches are at present perforce combined, or suggest some other way out of the difficulty, there would be some point to their remarks, but until then no doubt that heroic soul, the District Nurse, Reg. C.M.B., as the advertisement puts it, will go on her way quietly doing a noble work which few people see, spending a great deal of her time on her bicycle, going miles between each patient, out in all weathers night and day, giving skilled assistance to the young mother here and kindly attention to the bedridden old body there, in spite of our deeply rooted up-to-date notions that it should not be. . . . Can anyone find a remedy without appealing to the rates?

[The combination may conceivably, in a sparsely inhabited district, be a necessity, but even then it is a bad one, and should be avoided if possible.—Ed. The Hospital.]

### A WEAK SPOT IN GIRLS' EDUCATION.

"E. M. M." writes: In these days when no trouble is spared in the education of upper and middle-class girls to prepare them to fill their places in the world, one subject still remains in which it is almost impossible for them to get any instruction at all—namely, the care in health and sickness of babies and young children. Lucky are the girls who are blessed with brothers and sisters several years younger than themselves! They almost unconsciously learn how babies should be treated, and are in great measure spared the bitterness of buying their own experience later, at the expense too often of losing their first child. Some mothers are lucky enough to secure a "treasure" of a nurse, but they are few in number, and illness or home trouble is likely at any moment to deprive the child of her care, then if the mother is ignorant there is serious risk and much sorrow. A day spent in the out-patient department of one of our large London hospitals is a splendid object lesson, showing that errors of diet and clothing, not inherited disease, are the cause of two-thirds of the illness in the children who come before the doctors. Would it be feasible for some instruction to be given in connection with the children's hospitals which would be of great benefit to the girls and young married women, and at the same time assist the funds? If a member of the medical staff, or the Matron, could be persuaded to give a course of six simple lectures, for which many people would gladly pay a guinea, there would be a handsome addition to the funds of the hospital after deducting the lecturer's fees. Many girls are most anxious to learn something of practical nursing, but now that three years' training has become the rule in most hospitals their doors are closed to the paying probationer who used to go for six months, and was exempt from some of the sweeping, brass cleaning, etc. Could the Matrons of some of the children's hospitals be persuaded to admit a few lady pupils who would pay £1 per week for the privilege of coming every day from 10 till 6? The girls would learn a great deal by helping the nurses and assisting in preparing foods for the babies, feeding and dressing some of the convalescent children, but not interfering or competing in any way with the probationers. They would cost the hospital nothing except the midday dinner, and would be there to assist the sister of the ward when the nurses who begin work at 7 are off duty. For a girl who was going abroad the experience would be most valuable, and probably many a grateful subscription would be the result.

### TRAVEL NOTES AND QUERIES.

By our Travel Correspondent.

Probationer at Seventeen Years of Age (Poppy).—Your inquiry does not belong to this column. Write to the Editor, and the answer will appear on the last page of the Nursing Section.

Sanatorium in Switzerland (E. C. H.).—Your question does not belong to this column. See answer to "Poppy." I fear there is nothing very cheap in Switzerland of the kind needed for your friend. There is a sanatorium called the Schatzalp, at Davos-Platz, but I fear it is far from suitable as to terms.

Convalescent Home in the South of France (A. P.).—I know of no home exactly such as you wish, but here are some addresses to which you might write. They are homes where ladies of very limited means are received: Villa Emily, at San Remo. Terms per week, doctor included, 25s. You must apply for entrance through Messrs. Barnett and Co., 67 Lombard Street, E.C. Then there is another home on the same lines, Villa Helvetia Garavan, Mentone, also 25s. per week, but doctor not included, I think. At neither of these homes are ladies received who need nursing or special assistance. If you can afford to pay as much as 4s. 2d. per day I can give you some other addresses if you let me know.

**Rules in Regard to Correspondence for this Section.—All questioners must use a pseudonym for publication, but the communication must also bear the writer's own name and address as well, which will be regarded as confidential. All such communications to be addressed "Travel Correspondent, 28 Southampton Street, Strand." No charge will be made for inserting and answering questions in the inquiry column, and all will be answered in rotation as space permits. If an answer by letter is required, a stamped and addressed envelope must be enclosed, together with 2s. 6d., which fee will be devoted to the objects of "The Hospital" Convalescent Fund. Ten days must be allowed before an answer can be published.**

### A Book and its Story.

### THREE SISTERS AND A BROTHER.

Miss Carey's new novel differs in no way from others that she has written. It maintains the same simple standard which does not pretend to reach higher than the level of everyday people and their doings. But her characters are always natural, and, as in the present book, they are people whom one meets in real life, and not overdrawn, highly-coloured caricatures.

The household of Peter Holt comprised himself, as head, and three sisters devoted to their brother, who in return was not insensible to their devotion nor affected by the comments of the not altogether disinterested circle that formed the matron's council in the little town of Abbey Thorpe, where he had just established himself in practice when the story opens. He was still living on in the old house that had been the home of the Holts since they were children. "The Red House was nearly opposite St. Andrew's Church across the wide boulevard. . . . The outlook was singularly quiet and peaceful, and hallowed by the thought of the dear ones laid to rest in the shady corner behind St. Andrew's. The Red House was by no means an attractive-looking abode to a stranger; it strongly resembled the house a child always draws on its slate—three steps and a door, with a narrow window on one side and two equally narrow ones on the other, and four upper ones placed at equal distances above them, topped by an attic or two. . . . However disappointing the frontage might be, the drawing-room or sitting-room, as it was called, where the girls spent all their time, was a charming old-fashioned room and a perfect nest of comfort. . . . Peter, who had just attained his thirtieth year, was considerably older than his three sisters."

Abbey Thorpe having its full complement of medical men, did not welcome the advent of an addition. Peter had to encounter some opposition, and made his way slowly, in spite of ability, for he excited the ire of the older practitioners, whose easy-going methods were in strong contrast to his own. "It must be owned that Dr. Holt was not a favourite with his professional colleagues. The elder men, who expectea due deference from their junior, were affronted by the young man's independence and audacity; he had a habit of sticking to his opinion, and maintaining it in an aggressive way that secretly excited their wrath. . . Dr. Holt was clever and enterprising, but the old-fashioned practitioners who drove through St. Andrew's Street in their softly-padded carriages, looked askance at the tall, broad-shouldered young man who walked so quickly, with his chin a little raised, and carrying his head high, as though he knew he had the world to conquer, and would shrug their shoulders a little disparagingly at Peter's w[illegible]rm enthusiasm and advanced theories." Peter's advanced theories were not confined to professional ones. He held others which, socially, were not in accordance with those of his sisters. The book begins with a scene in which a discussion has arisen between them in consequence of his having requested them to call upon Miss Burke, who had, until the death of Mrs. Wallace, whom Peter had attended, filled the post of housekeeper to her. Peter having recognised the sterling qualities which Hannah Burke possessed, was anxious that they should make her acquaintance. Mrs. Wallace had acknowledged her indebtedness for ten years' faithful attendance in the do[illegible]ble capacity of nurse and housekeeper, by leaving her the house, the furniture, and a comfortable income.

Mrs. Wallace was eccentric and full of cranks, but she was kindhearted, and Hannah Burke was sincerely attached to her. Dr. Holt, observing the patience and tact exercised by Hannah towards his trying patient, had suggested that she should take up nursing as a profession. But when the contents of the will were known it was no longer necessary for her to consider the idea, although throughout the story she was constantly giving him assistance gratuitously, when he called for her aid in an emergency. When, after some hesitation on the part of his sisters, they decide to call at "Wayside," the house of which Hannah was now mistress, we read that the girls were in no hurry to reach their destination, and the charms of the scene before them were lost to them, for they had undertaken the expedition solely out of deference to Peter's request, and entirely in opposition to their own inclination. "If we have Sahara in our hearts, the landscape at our feet may be like the Land of Beulah, and yet give us no pleasure. Wayside was really a charming house. It was a low, grey cottage, clothed with creepers almost to the chimney stacks. It had a wide deep porch full of hanging baskets and plants, and a pretty old-fashioned garden lay round it. As the sisters walked up the path they had a glimpse of a sunny lawn, with beehives and a sundial, and a rustic seat under a tree. 'Oh! Vera, if only someone nice lived here!' ejaculated Ranee, as she rang the bell." The girls were received by Hannah in the porch. They were charmed with the interior of Wayside, with its air of dignified, old-world repose and refinement, all speaking of a vanished age, in which culture and taste were conspicuous. "The furniture was charmingly antique. There were delightful old Chippendale cabinets full of china and curious shells, one or two spindle-legged tables, and a spinning-wheel, and a harp added to the picturesque effect, and even the stiff-backed couch seemed in harmony with the whole. There was nothing modern." The only incongruous thing was the presence of Hannah Burke, who is described as seeming curiously out of place in her environment. "Now Hannah Burke was essentially modern. She looked the incarnation of practical everyday utility and common sense in her black stuff dress, over which she wore a white linen apron with a bib, as though to protect her mourning. Vera said afterwards that if she had worn a cap she would have looked like a nursing sister, with her deep linen cuffs and collar. . . Miss Burke's paleness seemed normal, but her complexion was perfectly clear and healthy, and the reddish-brown hair looked almost copper coloured in the sunshine. There was something pleasing, too, in her expression, and the straightforward look of her grey eyes; and there was no doubt that Peter was right in recommending her to their kindness." Hannah Burke stands out prominently at the opening and the close of the story of Peter's Household, but the intervening parts of the book have to do with other and very different characters. A bicycle accident brings the daughter of a neighbouring squire as a temporary visitor and patient to Peter and his sisters. From this other incidents arise in which their future is involved. Ranee Holt accompanies the patient, Alise Weston, home when she is well enough to be moved, and from this time their circle widens beyond the limits of the little town of Abbey Thorpe. A secret marriage is the one tragic element in an otherwsie unexciting story. But the picture of Peter's sisters in their home life, if sometimes a little drawn out, is both touching and interesting.

* "The Household of Peter." By Rosa N. Carey. (Macmillan and Co. 6s.)

### Notes and Queries.

**REGULATIONS.**

**The Editor is always willing to answer in this column, without any fee, all reasonable questions, as soon as possible.**

**But the following rules must be carefully observed.**

**1. Every communication must be accompanied by the name and address of the writer.**

**2. The question must always bear upon nursing, directly or indirectly.**

**If an answer is required by letter a fee of half-a-crown must be enclosed with the note containing the inquiry.**

*Visiting Nurse in Marylebone.*

(43) Can you tell me the name of the nurse who first started "visiting" in Marylebone?—*R. A.*

Write to the Hon. Secretary of the Marylebone Association, 126 Seymour Place, Marylebone, W.

*Medico-psychological Certificate.*

(44) I have just finished twelve months training in a naval hospital in general work, and should like to enter some mental asylum to qualify for the Medico-psychological certificate. Can you advise me where to apply and how to proceed?—*E. J. F.*

You can prepare for the examination in any of our large lunatic asylums. See list in Burdett's "Hospitals and Charities," or write to the Medico-Psychological Association of Great Britain and Ireland, 11 Chandos Street, London, W.

*Library for Medical Books.*

(45) Is there any library where one can obtain medical books either free or by a small yearly payment?—*W. E. J.*

Messrs. Lewis have a medical circulating library in Gower Street.

*Asylum.*

(46) Will you inform me what asylums would receive a young gentlewoman (mental) for fees of little more than £100 a year, and would treat her with patients of her own class?—*Misericordia.*

You will find a list of suitable private asylums in Burdett's "Hospitals and Charities," or advertise, and be careful to have references from the friends of present patients before deciding. Most of the County Asylums receive private patients at less expense.

*Funeral Expenses and Duties.*

(47) Are the parents responsible for the funeral expenses of a nurse who died abroad while nursing cholera? Also if a nurse can be compelled to take up her duties until 12 noon on the day she is leaving the institution?—*D. M.*

It is impossible to answer your first question without fuller particulars, but a nurse would be expected to fulfil her duties up to 12 noon on the day she leaves the institution.

*Central Midwives Board.*

(48) Will you be so good as to give me the address of the Secretary of the Central Midwives Board?—*Clinicus.*

6 Suffolk Street, Pall Mall, S.W.

*Boys' School.*

(49) Please tell me if there be an educational agency, or what would be the best way to secure a matron's post in a boys' school?—*Gran.*

Watch our advertisement columns and those of the educational papers.

*Midwifery.*

(50) I should be glad to know if the certificate of maternity nursing and gynæcological nursing of the British Gynæcological Society would be of any service, either now or in the future, to a fully-trained nurse holding the L.O.S. certificate?—*Inquisitive.*

The L.O.S. is in itself enough, subject to the holder having also satisfied the Central Midwives Board. To anyone so equipped the additional certificates you name would be of the same value that the L.S.A. would be to a fully-qualified medical man.

**Handbooks for Nurses.**

| | Post Free. |
|---|---|
| "A Handbook for Nurses." (Dr. J. K. Watson.) ... | 5s. 4d. |
| "Nurses' Pronouncing Dictionary of Medical Terms" | 2s. 0d. |
| "Art of Massage." (Creighton Hale.) ... ... ... | 6s. 0d. |
| "Surgical Bandaging and Dressings." (Johnson Smith) | 2s. 0d. |
| "Hints on Tropical Fevers." (Sister Pollard.) ... | 1s. 8d. |

Of all booksellers or of The Scientific Press, Limited, 28 & 29 Southampton Street, Strand, London, W.C.

### For Reading to the Sick.

"THE MEEK SHALL INHERIT THE EARTH."

Oft in life's stillest shade reclining
In desolation unrepining
Without a hope on earth to find
A mirror in an answering mind.
Meek souls there are who little dream
Their daily strife an angel's theme,
Or that the rod they take so calm
Shall prove in heaven a martyr's balm.

*Keble.*

We remember how David says "The meek-spirited shall possess the earth: and shall be refreshed in the multitude of peace"; or how the Son of David says "Blessed are the meek: for they shall inherit the earth." Or again, "Learn of Me; for I am meek and lowly in heart." Or again, how it is written "Them that are meek shall He guide in judgment." And we note the particular blessing which is attached to the possession of this virtue, both in the Old Testament and in the New; it is the privilege of the meek that they shall inherit the earth. Those who are always being pushed to the wall, those who do not assert themselves, who are retiring, who are put upon; these are just the men who shall inherit the earth.

Oh! how much good there is in the world! Let us remember this. It was said in one of those revolutionary disturbances which from time to time have broken over Paris, that when "the party of order" had the courage to take to the streets, they were surprised to find how many they were; if we could see the good that is going on all around us, it would not only cheer us, but make us humble. Those who are moving up and down among the wounded in life's conflict, to heal, to cheer, and to soothe, are not so conspicuous as the glitter and glare of arms and accoutrements, and the flash and gleam of battle. The grand ship cuts her way through the waves, with swift and powerful motion, and we do not stop to think of those who are working out of sight to secure that motion. The strength and beauty of life around us is owing, it may be, to those whose left hand does not know what their right hand is doing.

And so we might hope that, living in God's presence, knowing ourselves, and honouring all men, we might, out of the flower of humility, develop that blessed fruit of Meekness, which surely is one of the Holy Spirit's choicest gifts.

*Canon Newbolt.*

O Father! not my will but
Thine be done,
So spake the Son.
Be this our charm mellowing
Earth's ruder noise
Of griefs and joys:
That we may cling for ever to
Thy breast
In perfect rest.

*Keble.*

## Figures and Tables

**Figure f1:**
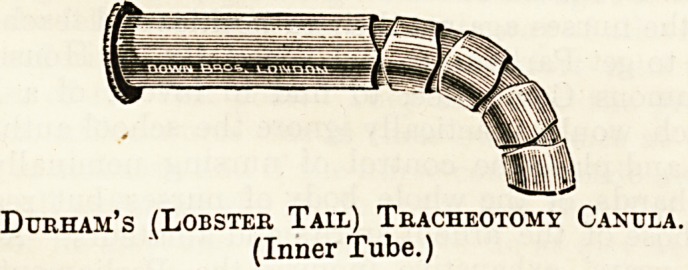


**Figure f2:**
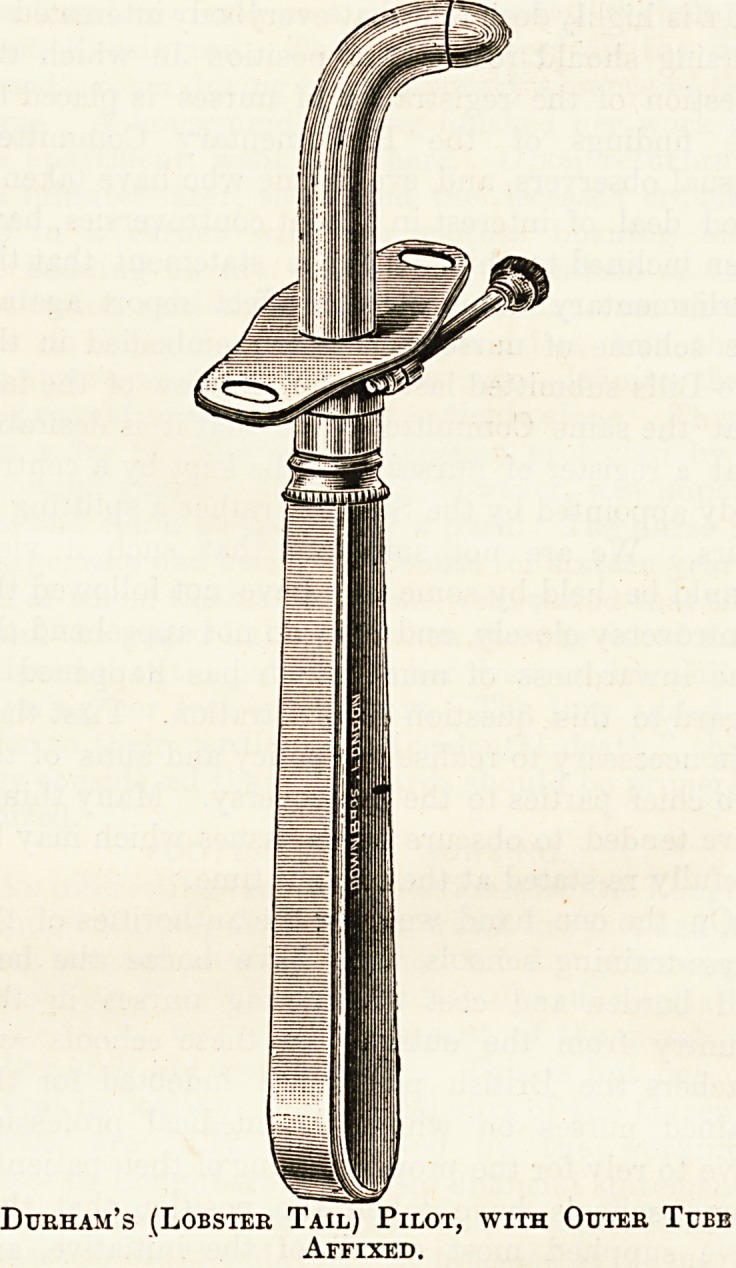


**Figure f3:**
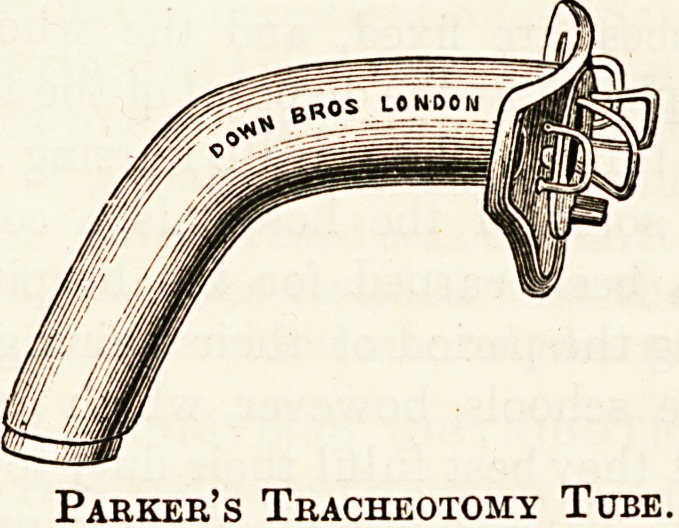


**Figure f4:**